# Component Asymmetry in Robotic-Assisted Bilateral Total Knee Arthroplasty

**DOI:** 10.7759/cureus.101056

**Published:** 2026-01-07

**Authors:** Taiceer Abdulwahab, Subhashree Ravi, Hesham Alkhateeb, Ahmed Elhemaky, Karl F Almqvist, Saeed Althani, Ali Albelooshi

**Affiliations:** 1 Orthopaedic Surgery, King's College Hospital London, Dubai, ARE; 2 Trauma and Orthopaedics, Mediclinic City Hospital, Dubai, ARE; 3 Surgery, Hull York Medical School, York, GBR; 4 Orthopaedic Surgery, Emirates Specialty Hospital, Dubai, ARE; 5 Orthopaedic Surgery, Mediclinic Parkview Hospital, Dubai, ARE; 6 Orthopaedics, Mediclinic City Hospital, Dubai, ARE; 7 Orthopaedic Surgery, Orthocure Medical Center, Dubai, ARE

**Keywords:** component alignment, knee arthroplasty, orthopaedic navigation, primary knee replacement, robotic knee surgery

## Abstract

Introduction

Accurate sizing of femoral and tibial components is fundamental to achieving successful outcomes in total knee arthroplasty (TKA). Oversizing can increase patellofemoral contact forces and restrict flexion, whilst undersizing may result in instability. Although conventional TKA is well established, robotic-assisted systems provide enhanced alignment accuracy, individualised planning, and reduced periarticular soft-tissue injury. Bilateral TKA is increasingly performed in Middle Eastern populations, where knee anthropometry differs from Western cohorts. Previous studies in conventional bilateral TKA have demonstrated notable asymmetry between femoral and tibial components. The aim of this study was to determine the frequency of component asymmetry in simultaneous bilateral robotic-assisted TKA using the Navio robotic system in a Middle Eastern population.

Methods

A retrospective review was undertaken of all patients undergoing primary, uncomplicated simultaneous bilateral robotic-assisted TKA at Mediclinic City Hospital, Dubai, UAE, between January 2018 and January 2020. Only procedures performed by a single surgeon were included. Demographic data, body mass index (BMI), and component sizes were recorded. All procedures utilised implants from the Anthem Total Knee System (Smith & Nephew, London, UK), designed with standard and narrow femoral options to minimise medio-lateral overhang. Symmetry of femoral, tibial, polyethylene, and patellar components was assessed. Standard perioperative protocols for infection prevention, thromboprophylaxis, and enhanced recovery rehabilitation were employed.

Results

A total of 140 patients (78.6% female patients, mean age 70.4 ± 8.8 years) were included. The mean BMI fell within the overweight to obese range. Femoral components were identical in 132 patients (95%), with asymmetry present in eight patients (5%). Of these, six patients (4%) demonstrated a one-size difference, and eight exhibited variation in femoral component width (standard versus narrow). All tibial baseplates, polyethylene inserts, and patellar components were symmetrical across both knees (100%).

Conclusion

This is the largest series to date reporting on component symmetry in robotic-assisted bilateral TKA in a Middle Eastern population. The findings demonstrate a high degree of femoral and tibial component symmetry, with only 5% of patients requiring femoral asymmetry and none requiring tibial or patellar asymmetry. Compared with prior reports of conventional bilateral TKA in similar populations, robotic-assisted TKA showed markedly lower asymmetry rates. These results suggest that robotic technology improves the accuracy of component sizing and alignment, reduces mismatch rates, and may enhance long-term functional outcomes. Further prospective studies with functional and radiological endpoints are recommended to corroborate these findings and explore cost-effectiveness in the wider clinical practice.

## Introduction

Total knee arthroplasty (TKA) is among the most frequently performed orthopaedic procedures. Achieving an accurate fit of femoral and tibial components is essential to optimise functional outcomes and minimise postoperative complications [1]. Oversizing of the femoral component has been associated with increased patellofemoral contact pressures, decreased flexion gap, and reduced postoperative range of motion [2]. In a study by Mahoney et al., oversizing by ≥3 mm was correlated with greater postoperative knee pain [3].

In conventional TKA, surgical accuracy can be limited by instrumentation constraints, potentially resulting in malalignment and suboptimal implant positioning. While conventional TKA is safe and effective [4], robotic-assisted systems aim to enhance precision by integrating preoperative and intraoperative data to generate patient-specific 3D models. These allow for refined bone resection and implant placement while accommodating individual ligament balance and lower limb alignment [5-8].

In the Middle East, the prevalence of advanced knee arthritis requiring TKA is high [9,10], and the frequency of bilateral TKA is increasing. Sivaram et al. [11] reported notable rates of femoral and tibial component asymmetry in staged bilateral TKA in this population. This study aims to measure the frequency of femoral and tibial component asymmetry in simultaneous bilateral robotic-assisted TKA performed at a single tertiary centre in Dubai, UAE.

This article was previously presented as a meeting poster presentation at the European Federation of National Associations of Orthopaedics and Traumatology (EFORT), Lisbon, Portugal in June 2022.

## Materials and methods

Study design and setting

This was a retrospective observational study of patients undergoing simultaneous bilateral robotic-assisted total knee arthroplasty (TKA) at Mediclinic City Hospital, Dubai, United Arab Emirates. The study period was January 2018 to January 2020.

Participants

All patients who underwent primary, uncomplicated simultaneous bilateral robotic-assisted TKA during the study period were eligible. Inclusion criteria were: (i) simultaneous bilateral procedures; (ii) surgery performed by a single surgeon (A.B.); and (iii) complete data available in the hospital information system and implant registry. Patients operated on by more than one surgeon were excluded to eliminate inter-surgeon variability (Table 1).

**Table 1:** Inclusion/Exclusion criteria TKA = Total Knee Arthroplasty

Criteria type	Criteria
Inclusion	Simultaneous bilateral robotic-assisted TKA
	Primary, uncomplicated cases
	Single-surgeon procedures
Exclusion	Cases involving different surgeons for each knee
	Complex or revision procedures

Data sources and variables

Data were obtained from the hospital electronic records and verified against the operating theatre implant registry. Variables collected included age, sex, body mass index (BMI), and implant sizes (femoral, tibial, polyethylene, patellar).

Preoperative protocol

All patients underwent standard preoperative evaluation, including blood tests, radiographic imaging, and Methicillin-resistant Staphylococcus aureus (MRSA) screening with nasal, axillary, and groin swabs. Routine enhanced recovery protocols were followed [12].

Operative technique

Procedures were performed under spinal anaesthesia and tourniquet control through a medial parapatellar approach with patellar eversion. The Navio Robotic System (Smith & Nephew, London, UK) was used for intraoperative patient-specific planning, creating a three-dimensional model of the knee to assess ligament balance, component alignment, and resection accuracy.

Femoral sizing was performed using posterior referencing jigs with 30° external rotation. For borderline (“mid-lier”) cases, the larger size was selected to avoid anterior cortical notching [13]. Tibial preparation was performed using extra-medullary alignment jigs, referencing the tibial crest and the second toe. Osteophytes were excised circumferentially to avoid component overhang.

Implants

All patients received the Anthem Total Knee System (Smith & Nephew, London, UK), which provides both standard and narrow femoral options for each anteroposterior size, reflecting anthropometric adaptations for Middle Eastern knees [14].

Perioperative management

Patients received three doses of first-generation cephalosporin for infection prophylaxis and subcutaneous enoxaparin for two weeks post-operatively. Early mobilisation began on post-operative day one with a walking frame. Rehabilitation was guided by an enhanced recovery programme [15].

Outcome assessment

Implant symmetry was assessed by comparing component sizes (femoral, tibial, polyethylene, patellar) between left and right knees. Radiographs obtained post-operatively confirmed component placement and limb alignment.

Data analysis

Data were entered into a secure database and analysed using IBM SPSS Statistics for Windows, Version 25 (Released 2017; IBM Corp., Armonk, New York, United States). Continuous variables (age, BMI) were summarised as mean ± standard deviation (SD). Categorical variables (sex, component symmetry/asymmetry) were expressed as frequencies and percentages. The primary outcome was the proportion of patients with component asymmetry in femoral and tibial implants. No formal hypothesis testing was undertaken as the study was descriptive in nature.

## Results

Demographic data

A total of 140 patients underwent simultaneous bilateral robotic-assisted TKA during the study period (January 2018-January 2020). The majority were female patients (n=110, 78.6%). The mean age was 70.4 years (SD ±8.8, range 48-92). The mean BMI was 29.7 ± 4.6 kg/m². Patient demographics are summarised in Table 2.

**Table 2:** Demographic data BMI = Body Mass Index

Variable	Value
Total patients	140
Sex (F:M)	110:30 (78.6% female patients)
Age (years)	Mean 70.4 ± 8.8 (range 48–92)
Mean BMI (kg/m²)	29.7 ± 4.6

Femoral components

In 132 patients (95%), identical femoral component sizes were implanted bilaterally. Asymmetry was identified in eight patients (5%; Table 3).

**Table 3:** Component symmetry in bilateral robotic-assisted total knee arthroplasty

Component	Symmetrical, n (%)	Asymmetrical, n (%)	Details of asymmetry
Femoral component	132 (95%)	8 (5%)	One-size difference in six patients; Width difference (standard vs narrow) in eight patients
Tibial component	140 (100%)	0 (0%)	None
Polyethylene insert	140 (100%)	0 (0%)	None
Patellar component	140 (100%)	0 (0%)	None

Size discrepancy

Six patients (4%) demonstrated a one-size difference between the two knees. The maximum variation in femoral component size was a single increment; no cases exceeded this difference.

Width discrepancy

Eight patients required differing femoral component widths, with one knee receiving the narrow version and the contralateral knee receiving the standard version of the same anteroposterior size.

Tibial components

All 140 patients (100%) received identical tibial baseplates bilaterally. No size or width mismatches were recorded.

Polyethylene inserts

Polyethylene insert sizes were symmetrical in all patients (100%).

Patellar components

Patellar component sizes were identical across all patients (100%).

Overall component symmetry

When considering all components, the overall frequency of asymmetry was 5%, confined exclusively to the femoral components. No tibial, polyethylene, or patellar asymmetry was observed. Implant symmetry data are summarised in Table 3.

Postoperative imaging and early outcomes

Immediate postoperative radiographs confirmed correct alignment and component positioning in all patients. No intraoperative complications or cases of early revision were recorded within the initial hospital stay. The mean length of stay was three to four days, consistent with the institutional enhanced recovery pathway.

## Discussion

This study found a 5% rate of femoral component asymmetry in simultaneous bilateral robotic-assisted TKA, with complete symmetry in tibial, polyethylene, and patellar components. These results compare favourably to those reported by Sivaram et al. [11] in a Middle Eastern cohort undergoing conventional bilateral TKA, where femoral and tibial asymmetry rates were 34.1% and 24.4%, respectively. Similar findings have been reported by Brown et al. [15], Capeci et al. [16], and Reddy et al. [17], who demonstrated variable rates of femoral and tibial asymmetry in conventional bilateral procedures, highlighting the challenges of achieving accurate component sizing without robotic assistance.

Robotic-assisted TKA offers stereotactic boundaries and intra-operative three-dimensional modelling, which enhance the precision of bone resections and implant positioning [5-8]. This improved accuracy likely explains the low incidence of asymmetry in the present series. Our findings suggest that robotic workflows may reduce component mismatch rates, which are associated with altered biomechanics, reduced flexion-extension gap balance, and increased joint contact forces [2,3,11]. In particular, correct femoral sizing is critical, as oversizing can lead to patellofemoral overstuffing and restricted range of motion, whilst undersizing risks joint laxity and instability. Likewise, oversized tibial components may increase the risk of malrotation and soft-tissue impingement [11].

Our results also compare well with published data from robotic TKA series. Kayani and Haddad [6] reported that robotic systems reduce alignment outliers and improve gap balancing compared with conventional jig-based TKA. Song et al. [7] and Bellemans et al. [8] similarly demonstrated reduced variability in component positioning with robotic assistance. The present findings reinforce these observations, suggesting that robotic-assisted bilateral TKA can reliably achieve component symmetry, particularly in populations with anatomical variations such as Middle Eastern knees [11].

From a clinical perspective, maintaining symmetrical component sizing in bilateral TKA is essential to ensure balanced flexion-extension gaps, optimal patellofemoral tracking, and long-term implant function. Even small discrepancies can influence joint kinematics and patient satisfaction. Our study suggests that robotic systems can minimise such mismatches, potentially translating into improved functional outcomes, faster rehabilitation, and enhanced patient-reported satisfaction. However, longitudinal studies are required to confirm whether these technical advantages result in improved survivorship and reduced revision rates.

Limitations

This study has several limitations. First, it is retrospective in design and limited to a single-centre experience with one operating surgeon, which may restrict generalisability. Second, the analysis was descriptive, and no functional or patient-reported outcome measures were included, preventing correlation of component symmetry with clinical outcomes. Third, the absence of a direct comparison group undergoing conventional bilateral TKA within the same institution limits the strength of comparative conclusions. Finally, while robotic systems improve accuracy, femoral sizing decisions retain a subjective element, particularly in borderline cases.

## Conclusions

This study demonstrates that in simultaneous bilateral robotic-assisted TKA using the Navio surgical system, the vast majority of patients received identical femoral component sizes, with complete symmetry observed in all tibial baseplates, polyethylene inserts, and patellar components. The low incidence of femoral component asymmetry observed here is notably less than that reported in studies of conventional bilateral TKA in similar populations, supporting the premise that robotic assistance enhances intra-operative precision in component sizing and placement.

Robotic-assisted TKA offers several clear advantages over conventional jig-based techniques, including enhanced accuracy in bone resections, improved restoration of mechanical alignment, and reduced soft tissue trauma. In our cohort, standardised operative techniques, meticulous preoperative planning, and consistent execution by a single experienced surgeon likely contributed to the high rate of component symmetry and the absence of major intraoperative complications. These factors, combined with the stereotactic control afforded by robotic guidance, appear to mitigate sizing errors that can otherwise result in joint instability, altered kinematics, and patient dissatisfaction.

From a clinical perspective, maintaining accurate and symmetrical component sizing in bilateral TKA is essential for optimising flexion-extension gap balance, patellofemoral tracking, and long-term prosthesis function. Even small mismatches in femoral or tibial sizing can alter joint biomechanics and potentially impact patient outcomes. Our findings suggest that robotic-assisted workflows can substantially reduce such mismatches, which may translate into improved function, faster rehabilitation, and greater patient satisfaction.

However, broader adoption of robotic-assisted TKA will require consideration of its limitations, including higher capital costs, the need for system-specific training, and potential procedural learning curves. Long-term comparative studies are needed to determine whether the precision benefits of robotic-assisted bilateral TKA translate into superior survivorship, reduced revision rates, and sustained functional gains over conventional approaches.

In summary, for appropriately selected patients undergoing simultaneous bilateral TKA, robotic assistance provides a safe, reproducible, and highly accurate method of achieving component symmetry, thereby addressing an important technical factor associated with long-term success in knee arthroplasty.

## References

[1] Mihalko W, Fishkin Z, Krackow K (2006). Patellofemoral overstuff and its relationship to flexion after total knee arthroplasty. Clin Orthop Relat Res.

[2] Kawahara S, Matsuda S, Fukagawa S (2012). Upsizing the femoral component increases patellofemoral contact force in total knee replacement. J Bone Joint Surg Br.

[3] Mahoney OM, Kinsey T (2010). Overhang of the femoral component in total knee arthroplasty: risk factors and clinical consequences. J Bone Joint Surg Am.

[4] Saber AY, Marappa-Ganeshan R, Mabrouk A (2023). Robotic assisted total knee arthroplast. StatPearls [Internet].

[5] Hampp EL, Chughtai M, Scholl LY, Sodhi N, Bhowmik-Stoker M, Jacofsky DJ, Mont MA (2019). Robotic-arm assisted total knee arthroplasty demonstrated greater accuracy and precision to plan compared with manual techniques. J Knee Surg.

[6] Kayani B, Haddad FS (2019). Robotic total knee arthroplasty: clinical outcomes and directions for future research. Bone Joint Res.

[7] Song EK, Seon JK, Yim JH, Netravali NA, Bargar WL (2013). Robotic-assisted TKA reduces postoperative alignment outliers and improves gap balance compared to conventional TKA. Clin Orthop Relat Res.

[8] Bellemans J, Vandenneucker H, Vanlauwe J (2007). Robot-assisted total knee arthroplasty. Clin Orthop Relat Res.

[9] Badsha H, Kong KO, Tak PP (2008). Rheumatoid arthritis in the United Arab Emirates. Clin Rheumatol.

[10] Dargham SR, Zahirovic S, Hammoudeh M (2018). Epidemiology and treatment patterns of rheumatoid arthritis in a large cohort of Arab patients. PLoS One.

[11] Sivaram R, Bandi A, Shanmugasundaram S, Hafez AT, Butt AJ, Al-Khateeb H (2020). Component asymmetry in bilateral total knee arthroplasty in the Middle Eastern population. J Arthrosc Joint Surg.

[12] McDonald DA, Siegmeth R, Deakin AH, Kinninmonth AW, Scott NB (2012). An enhanced recovery programme for primary total knee arthroplasty in the United Kingdom-follow up at one year. Knee.

[13] Zalzal P, Backstein D, Gross AE, Papini M (2006). Notching of the anterior femoral cortex during total knee arthroplasty characteristics that increase local stresses. J Arthroplasty.

[14] Ren Y, Cao S, Wu J, Weng X, Feng B (2019). Efficacy and reliability of active robotic-assisted total knee arthroplasty compared with conventional total knee arthroplasty: a systematic review and meta-analysis. Postgrad Med J.

[15] Brown TE, Diduch DR, Moskal JT (2001). Component size asymmetry in bilateral total knee arthroplasty. Am J Knee Surg.

[16] Capeci CM, Brown EC 3rd, Scuderi GR, Scott WN (2006). Component asymmetry in simultaneous bilateral total knee arthroplasty. J Arthroplasty.

[17] Reddy VG, Mootha AK, Thayi C, Kantesaria P, Kumar RV, Reddy D (2011). Are both the knees of the same size? Analysis of component asymmetry in 289 bilateral knee arthroplasties. Indian J Orthop.

